# Annotations

**Published:** 1908-08-08

**Authors:** 


### ANNOTATIONS.

### Workmen's Compensation Acts.

The knotty problems arising from the various Acts of Parliament, popularly included in the phrase "Compensation Acts," have already provided a good deal of work for the Courts and especially for the Appeal Courts. On one day last week three such appeals were heard, one of them in the House of Lords. From a medical jurisprudent's point of view the most interesting is that of Haylett *v.* Vigor, in which the liability of an employer was discussed for death due to granular kidney in a man who had previously suffered from lead poisoning. The words of the Act provide that "lead poisoning or its sequelæ" is a form of industrial disease for the results of which compensation is to be allowed; the County Court Judge found (1) that the immediate cause of death was granular kidney; (2) that granular kidney is a sequela of lead poisoning, but is also a sequela of gout, alcoholism, heart disease, and other complaints; and (3) that lead poisoning was not proved to be the cause of granular kidney in the deceased, but that the employers had not proved that it was not. On these findings he gave a verdict for the widow for £260. This judgment the Court of Appeal reversed on the ground that the onus of proof lies upon the workman, not on the employer. They held that the section quoted, when referring to a "sequela," means a consequence which is proved to be caused by the disease in question, not one which may have ensued from other causes. The wording therefore renders the section practically nugatory, for it is really impossible ever to prove conclusively what is the actual exciting cause of a chronic malady such as granular kidney; one may attribute it in any given case to gout, lead, or what not, with a fair show of reason; but that, of course, is not legal proof. Another point raised in the same case was as to the employer responsible; the deceased had worked as a painter for many years for one firm, and had changed his employment a month previously to the onset of symptoms. In this case it was not difficult to decide that the former firm was the real defendant to the action, but the line might, under other circumstances, be one not at all easy to draw.

### Livingstone College.

It was a happy inspiration which led the authorities of Livingstone College to invite Sir Patrick Manson to Leyton to deliver an address on the relation of tropical research to missionary enterprise. Anything more convincing of the necessity for and value of the College than Sir Patrick's brief sketch of his subject it would be hard to imagine; and this all the more so because the nature of missionary enterprise demands that its agents should live amongst the people whose endemic diseases are so fatal to Europeans. In times past, and even still to a considerable degree, it has been notorious that missionaries have displayed the most deplorable disregard for both common sense and science in their selections of sites for buildings and setlements, and in their views on hygiene: with the result that the mortality among them has been exceptionally and quite unnecessarily heavy. If this state of affairs is gradually passing away, to be replaced by care and attention to sanitary details which sometimes puts to shame even medical men, the credit for the improvement is very largely to be ascribed to the Livingstone College. In this institution are instructed men—clerical and lay—of all races and creeds in the peculiar dangers of the climates in which they work. The mere perusal of the names, religions, and destinations of those who have attended courses during the past year proves the success with which all such distinctions have been obliterated, and the wide recognition of the value of the health-training offered there. As each student signs a declaration that he will not assume the designation of medical missionary, we do not see that anything but good can come of the curriculum.

### Overlaying.

Hardly have the echoes of the Horsley-Troutbeck controversy died away before the coroner for South-West London is again in collision with a member of the medical profession. The facts, as reported in the press, seem to be that a Clapham practitioner was called at a quarter to seven one morning to an infant of ten weeks old, which had evidently been dead some hours. He gave a certificate of death from suffocation, and this was passed on to the coroner by the local registrar. At the inquest the pathologist whose name has been frequently before the profession in connection with these squabbles gave evidence that death was due to long-standing bronchitis, and the coroner in summing up made some strong remarks about the practitioner's conduct, as a consequence of which the latter gentleman was censured by the jury. Now, on this brief summary of the evidence, we should like to offer one or two remarks. First, a practitioner, we hold, puts himself in the wrong by giving a death certificate about a patient he never saw during life, and as to the manner of whose death there might be more than one opinion. Next, whenever Mr. Troutbeck wants to override a doctor's certificate of death he should employ a pathologist who enjoys the complete confidence of the medical profession; after the Horsley case one would have thought even he would be rather careful not to rely too implicitly upon post-mortem as opposed to clinical evidence. Again, as Dr. Atkinson has pointed out, a baby with bronchitis is stifled by a degree of restriction of its air supply which would hardly affect a healthy child; and, therefore, the truth in this case may well have been half-way between the extreme attitudes taken up by the doctor and the pathologist respectively. In the heat of the moment it apparently never struck anyone to inquire how it was that a tiny baby affected with longstanding bronchitis had received no medical care; an omission which, if that was the case, was far more culpable than accidental overlaying.

